# Intraoperative Neurophysiological Monitoring in Contemporary Spinal Surgery: A Systematic Review of Clinical Outcomes and Cost-Effectiveness

**DOI:** 10.3390/brainsci15070768

**Published:** 2025-07-19

**Authors:** Luca Zanin, Laura Broglio, Pier Paolo Panciani, Riccardo Bergomi, Giorgia De Rosa, Luca Ricciardi, Giusy Guzzi, Alessandro Fiorindi, Carlo Brembilla, Francesco Restelli, Francesco Costa, Nicola Montemurro, Marco Maria Fontanella

**Affiliations:** 1 Unit of Neurosurgery, ASST Spedali Civili di Brescia, 25123 Brescia, Italy; lucazanin00@gmail.com (L.Z.); rbergomi@tiscali.it (R.B.); alessandro.fiorindi@asst-spedalicivili.it (A.F.); marco.fontanella@unibs.it (M.M.F.); 2 Unit of Neurophysiopathology, ASST Spedali Civili di Bresciadi Brescia, 25123 Brescia, Italy; laura.broglio@gmail.com; 3 Neurosurgery Unit, Department of Medical and Surgical Specialties, Radiological Sciences and Public Health, University of Brescia, 25121 Brescia, Italy; g.derosa003@studenti.unibs.it; 4 UOC di Neurochirurgia, AOU Sant’Andrea, Dipartimento NESMOS, Sapienza University of Rome, 00185 Roma, Italy; ricciardi.lu@gmail.com; 5 Neurosurgery Department, “R. Dulbecco” Hospital, 88100 Catanzaro, Italy; guzzi.giusy@libero.it; 6 Department of Medical and Surgical Sciences, “Magna Graecia” University of Catanzaro, 88100 Catanzaro, Italy; 7 Department of Neurosurgery, IRCCS Humanitas Research Hospital, 20089 Rozzano, Italy; carlinobrembo@gmail.com; 8 Department of Neurosurgery, Fondazione IRCCS Istituto Neurologico C. Besta, University of Milan, 20133 Milan, Italy; francesco.restelli91@gmail.com (F.R.); francesco.costa@istituto-besta.it (F.C.); 9 Department of Neurosurgery, Azienda Ospedaliero Universitaria Pisana (AOUP), 56100 Pisa, Italy; nicola.montemurro@unipi.it

**Keywords:** intraoperative neurophysiological monitoring, technological innovation, spinal surgery, D-wave monitoring, economic analysis

## Abstract

**Background:** Intraoperative neurophysiological monitoring (IONM) is increasingly used during spinal surgery to reduce the risk of neurological complications. This systematic review evaluates both the clinical outcomes and cost-effectiveness of IONM in contemporary spinal surgery. **Methods:** A comprehensive literature search was conducted to identify studies evaluating IONM in spinal surgery. Twenty-three studies were included: twenty-one reporting clinical outcomes and two focusing on economic analysis. Data on neurological deficits, monitoring accuracy, and cost-effectiveness were extracted and analyzed. **Results:** Analysis of the included studies showed that IONM reduced the risk of neurological deficits across various types of spinal surgery. The diagnostic accuracy varied by modality, with MEP showing the highest sensitivity (90.2%) and SSEP demonstrating high specificity (97.1%). The greatest benefit was observed in deformity surgery and spinal tumors. D-wave monitoring showed efficacy for intramedullary tumors. Economic analysis demonstrated that IONM is cost-effective when the neurological complication rate exceeds 0.3%, with potential savings of over USD 23,000 per case. **Conclusions:** IONM significantly improves neurological outcomes in spinal surgery and is cost-effective in most clinical scenarios, particularly in high-risk procedures. Multimodal monitoring approaches provide the most comprehensive neurological assessment. These findings support the routine use of IONM in contemporary spinal surgery, especially for complex cases.

## 1. Introduction

Neurological injury remains one of the most devastating complications of spinal surgery, with potential for permanent disability and a significant impact on quality of life. Intraoperative neurophysiological monitoring (IONM) has emerged as a critical tool to detect neurological compromise during surgery, allowing for immediate intervention to prevent permanent damage [1,2,3]. Despite its widespread adoption, questions remain about its clinical efficacy and economic value in various spinal procedures [4].

This systematic review aims to provide a comprehensive evaluation of both the clinical outcomes and cost-effectiveness of IONM in contemporary spinal surgery. By analyzing data from twenty-three high-quality studies, including twenty-one focusing on clinical outcomes and two on economic analysis, we seek to provide evidence-based guidance for the use of IONM in different types of spinal procedures. The primary objectives of this systematic review are (1) to evaluate the efficacy of IONM in reducing neurological complications across different types of spinal surgery; (2) to assess the diagnostic accuracy of various IONM modalities; (3) to analyze the cost-effectiveness of IONM in spinal surgery; (4) to provide specific insights on the use of D-wave monitoring for intramedullary tumors; and (5) to examine the application of IONM in post-traumatic and osteoporotic vertebral fractures. This systematic review maintains a clear separation between clinical outcome analysis and cost-effectiveness evaluation, allowing for a comprehensive understanding of both aspects of IONM in contemporary spinal surgery.

## 2. Materials and Methods

### 2.1. Literature Search, Study Selection, and Data Extraction

**Figure 1:**
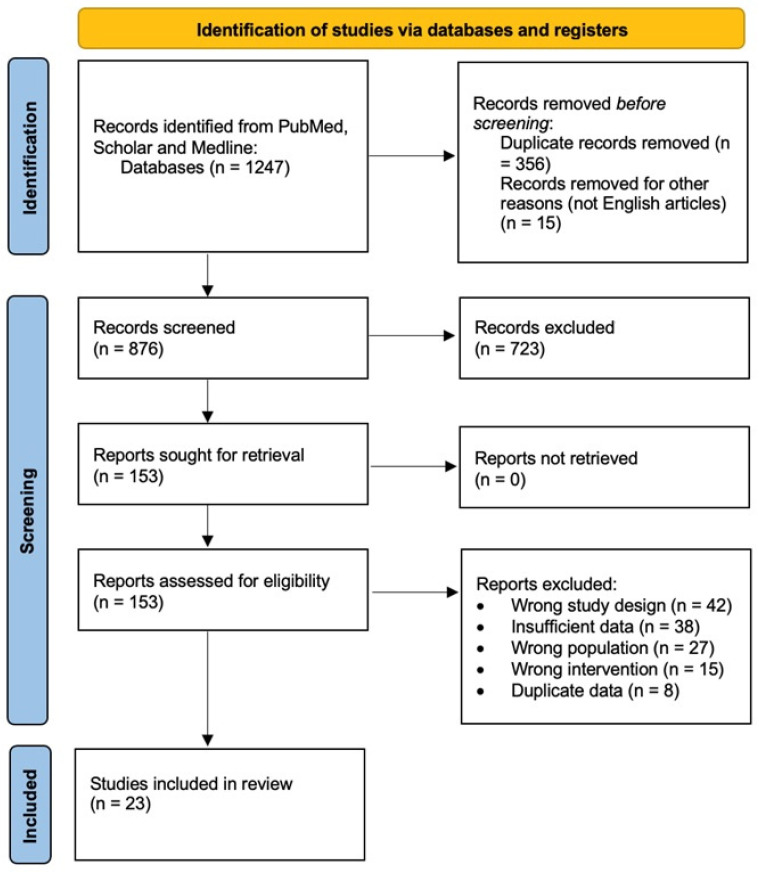
The PRISMA flow diagram illustrating the selection process of studies included in this systematic review.

A comprehensive literature search was conducted using the PubMed, Embase, and Cochrane databases to identify relevant studies published up to March 2025. Search terms included “intraoperative neurophysiological monitoring”, “IONM”, “spinal surgery”, “somatosensory evoked potentials”, “motor evoked potentials”, “D-wave”, “electromyography”, “cost-effectiveness”, and “economic analysis”. Studies were included if they (1) evaluated IONM in spinal surgery; (2) reported clinical outcomes, diagnostic accuracy, or economic data; (3) were published in English; and (4) included human subjects. Case reports, letters, and conference abstracts were excluded. Two independent reviewers extracted data from the included studies. For clinical studies, the following data were extracted: study design, sample size, type of spinal surgery, IONM modalities used, neurological outcomes, sensitivity, specificity, and follow-up duration. For economic studies, data on costs, savings, cost-effectiveness thresholds, and economic models were extracted. Twenty-three studies were included in this review, as the PRISMA flow diagram in Figure 1 shows [5].

### 2.2. Quality Assessment and Statistical Analysis

The quality of included studies was assessed using the Newcastle–Ottawa Scale for observational studies [6] and the Cochrane Risk of Bias Tool for randomized controlled trials [7]. Economic studies were evaluated using the Consolidated Health Economic Evaluation Reporting Standards (CHEERS) checklist [8]. For clinical outcomes, the relative risk reduction in neurological deficits was calculated. Pooled sensitivity and specificity were determined for diagnostic accuracy. For economic analysis, cost-effectiveness was evaluated based on reported savings, thresholds, and economic models. Subgroup analyses were performed based on the type of spinal surgery and IONM modalities.

## 3. Results: Clinical Outcomes

### 3.1. Study Characteristics

Of the 21 studies reporting clinical outcomes, there was a mix of study designs, including systematic reviews (Ajiboye et al., 2017; Alvi et al., 2024; El Choueiri et al., 2025; Thirumala et al., 2017) [9,10,11,12], retrospective analyses (Bai et al., 2025; Burkhard et al., 2025; Ibrahim et al., 2017; Kim et al., 2021; Raynor et al., 2016) [3,4,13,14,15], prospective studies (Calancie et al., 2014; Ilhan et al., 2024; Kim et al., 2025; Kundnani et al., 2010; Siller et al., 2024; Ushirozako et al., 2023; Yu et al., 2024) [2,16,17,18,19,20,21], observational studies (Cabañes-Martínez et al., 2024; CreveCoeur et al., 2024) [22,23], narrative reviews (Charalampidis et al., 2020; Guzzi et al., 2024) [24,25], and clinical practice guidelines (Fehlings et al., 2024) [26]. The total number of patients across all clinical studies was substantial, with several large-scale studies, including Alvi et al. (2024) [10] with 99,937 patients, El Choueiri et al. (2025) [11] with 187,162 patients, and Raynor et al. (2016) [4] with 12,375 patients. The types of spinal surgery included deformity correction, tumor resection, degenerative spine surgery, and trauma.

### 3.2. Efficacy of IONM in Reducing Neurological Complications

#### 3.2.1. Overall Efficacy

**Figure 2:**
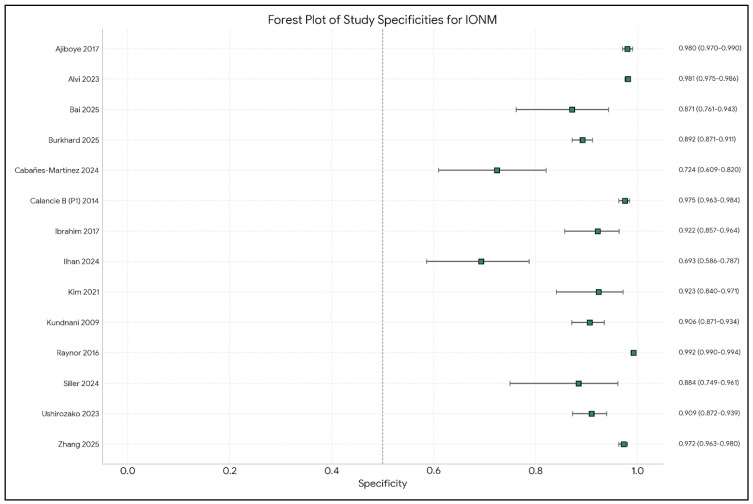
Forest plot of specificity estimates and 95% confidence intervals (CIs) for intraoperative neuromonitoring (IONM) in predicting neurological deficits, by study [1,3,4,9,10,13,14,15,16,17,19,20,21,22].

**Figure 3:**
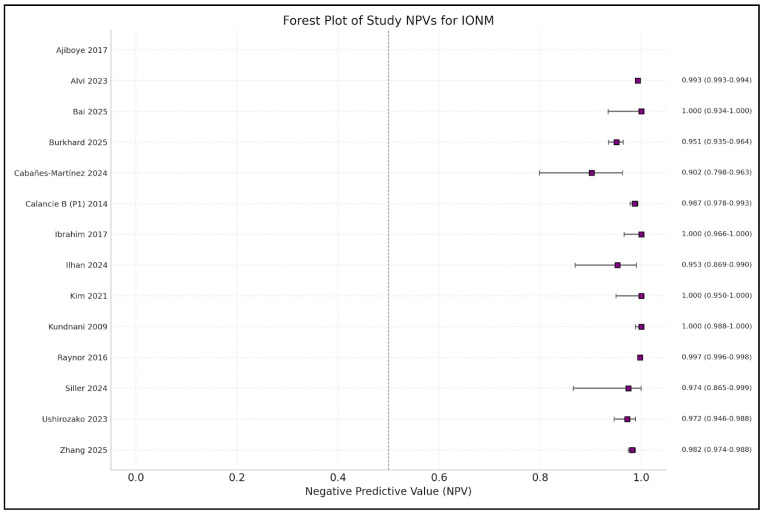
Forest plot of sensitivity estimates and 95% confidence intervals (CIs) for intraoperative neuromonitoring (IONM) in predicting neurological deficits, by study [1,3,4,9,10,13,14,15,16,17,19,20,21,22].

**Figure 4:**
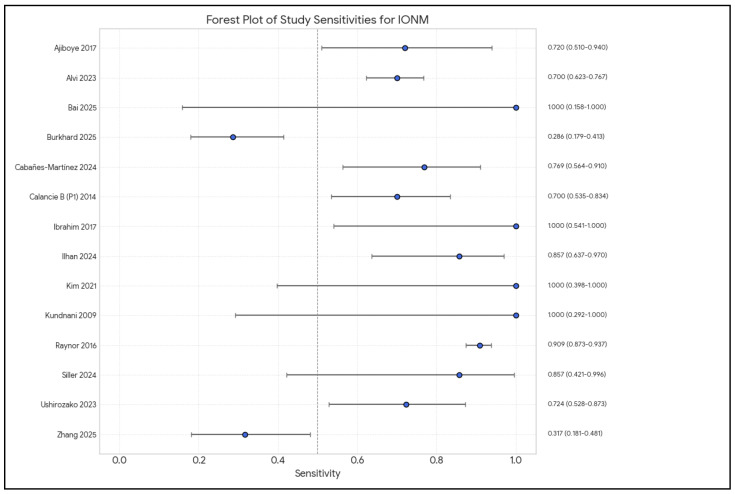
Forest plot of positive predictive value (PPV) estimates and 95% confidence intervals (CIs) for intraoperative neuromonitoring (IONM), by study [1,3,4,9,10,13,14,15,16,17,19,20,21,22].

**Figure 5:**
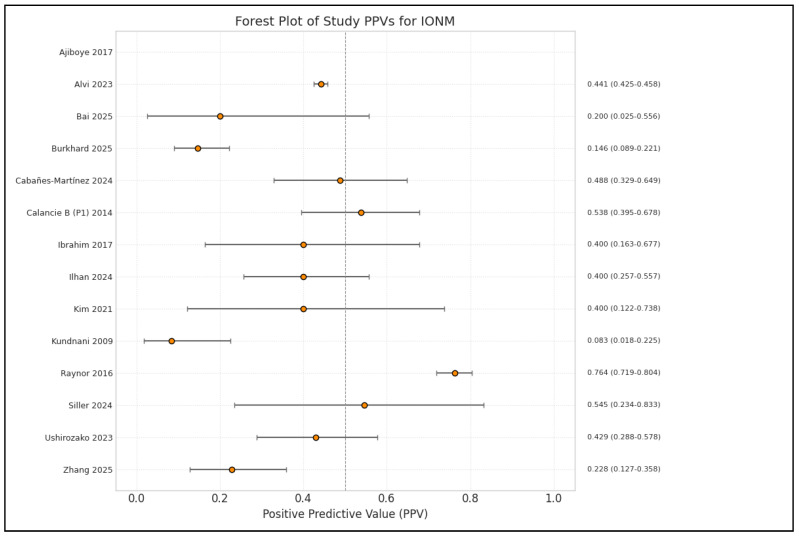
Forest plot of negative predictive value (NPV) estimates and 95% confidence intervals (CIs) for intraoperative neuromonitoring (IONM), by study [1,3,4,9,10,13,14,15,16,17,19,20,21,22].

**Figure 6:**
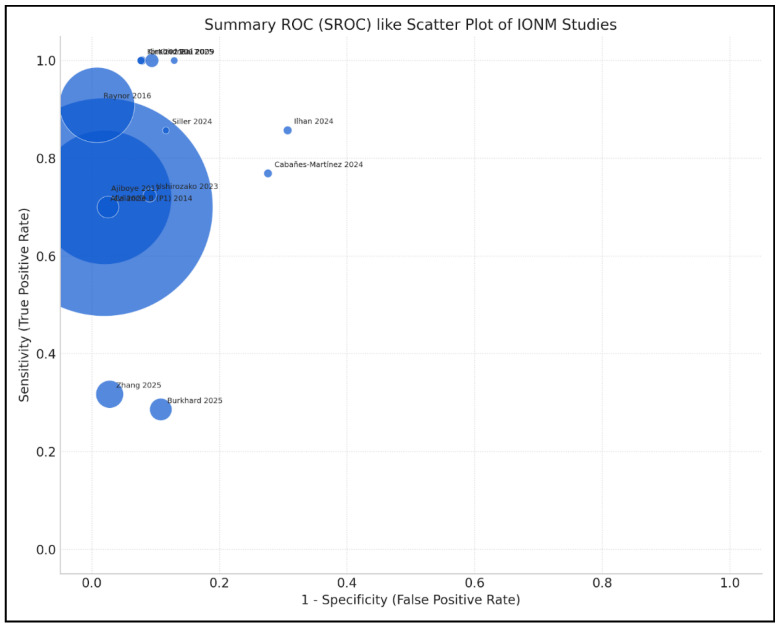
Scatter plot of sensitivity versus 1—specificity for included studies evaluating IONM diagnostic accuracy. Bubble size is proportional to the study’s sample size [1,3,4,9,10,13,14,15,16,17,19,20,21,22].

Analysis of the included studies showed varying efficacy of IONM in reducing neurological complications across different types of spinal surgery. The meta-analysis by Alvi et al. [10], which included 99,937 patients from 163 studies, provided comprehensive data on the diagnostic utility of different IONM modalities in detecting impending or incident intraoperative neurologic injuries. Figure 2, Figure 3, Figure 4, Figure 5 and Figure 6 show the results of the statistical analysis performed on the data of this systematic review.

#### 3.2.2. Efficacy by Surgery Type

*Deformity Surgery:* Ibrahim et al. [14] analyzed 121 patients and found that while IONM had a relatively low sensitivity (57.14%), it demonstrated high specificity (98.20%) in spinal deformity surgery. Kundnani et al. [19] reported that, in 354 patients with adolescent idiopathic scoliosis, neurogenic motor-evoked potentials (NMEPs) showed 100% sensitivity and 96% specificity, while somatosensory-evoked potentials (SSEPs) showed 51% sensitivity and 100% specificity.

*Tumor Surgery:* Ilhan et al. [17] studied 67 patients undergoing surgical resection of intradural spinal cord tumors and found that combined MEPs–D-wave/SSEPs had 95% sensitivity for detecting clinical deterioration at 3 months. Similarly, Cabañes-Martínez et al. [22] documented that, in 91 patients (36 monitored, 55 unmonitored), the rate of new/worsened neurological deficits was 8.3% in the monitored group versus 14.5% in the unmonitored group.

*Cervical Spine Surgery:* Ajiboye et al. [9] analyzed 26,357 patients from 10 studies and found that, for anterior cervical spine surgery (ACSS), IONM had a pooled sensitivity of 71% (CI: 48–87%) and specificity of 98% (CI: 92–100%). El Choueiri et al. (2025) conducted a meta-analysis of 187,162 patients (21,686 with IONM, 165,476 without IONM) from seven studies and found that, while pooled analysis showed no statistically significant protective effect of IONM in degenerative cervical spine surgery (OR = 0.90), subgroup analysis suggested a potential benefit when EMG is used with SSEP and MEP (OR = 0.39) [11].

*Complex Spinal Surgery:* Across various studies, multimodal IONM demonstrated significant value in complex spinal procedures. Yu et al. (2024) found, in their prospective, randomized, controlled study of 46 patients (23 IONM, 23 non-IONM), that IONM significantly reduced dysphagia 12 weeks post-anterior cervical spine surgery (13.0% vs. 43.5%) [2].

*Scoliosis Surgery:* Thirumala et al. [12] conducted a systematic review and meta-analysis of 2102 patients with idiopathic scoliosis from 12 studies and found that transcranial motor-evoked potential (TcMEP) monitoring had a pooled mean sensitivity of 91% (95% CI: 34–100%) and specificity of 96% (95% CI: 92–98%) for detecting new spinal cord injuries.

*Lumbar Spine Surgery:* Burkhard et al. (2025) studied 401 patients undergoing primary posterior lumbar interbody fusion (PLIF) and found that SSEP events were associated with postoperative motor deficits (*p* = 0.043) with SSEP events for PMDs showing 13.6% sensitivity and 96.0% specificity, while EMG events showed 36.4% sensitivity and 71.0% specificity [3].

#### 3.2.3. Diagnostic Accuracy of IONM, Multimodal Monitoring

The pooled analysis of studies reporting diagnostic accuracy showed varying results across different IONM modalities. According to Alvi et al. (2024), MEP showed the highest sensitivity at 90.2% (95% CI: 86.2–93.1), while SSEP demonstrated high specificity at 97.1% (95% CI: 95.3–98.3) [10]. EMG had lower sensitivity (48.3%, 95% CI: 31.4–65.6) but good specificity (92.9%, 95% CI: 84.4–96.9). Multimodal monitoring showed balanced performance with 83.5% sensitivity (95% CI: 81–85.7) and 93.8% specificity (95% CI: 90.6–95.9).

Multiple studies confirmed the superiority of multimodal intraoperative neurophysiological monitoring (MIONM) over single-modality monitoring. Fehlings et al. in their clinical practice guideline summarized the findings of Alvi et al. and recommended IONM for high-risk spine surgery patients (Strong recommendation, Low-quality evidence) [10,26]. Charalampidis et al. [24] noted in their narrative review that MIONM is generally effective for detecting perioperative neurological injury, though SSEP can be delayed, MEPs are sensitive to anesthesia, and EMG has a high false positive rate.

The efficacy of IONM is critically dependent on the anesthetic protocol employed, with total intravenous anesthesia (TIVA) significantly enhancing signal reliability compared with inhalational agents. Furthermore, a sophisticated tripartite communication process between the neurophysiologist, anesthesiologist, and surgeon is essential to differentiate true neurological compromise from false positive signals attributable to fluctuations in mean arterial pressure (MAP), significant blood loss, or anesthetic depth variations, thereby preventing unnecessary surgical modifications while ensuring genuine neurological alerts receive appropriate intervention.

#### 3.2.4. D-Wave Monitoring for Intramedullary Tumors

D-wave monitoring has emerged as a particularly valuable tool for intramedullary spinal cord tumor surgery. Siller et al. (2024) [20] conducted a prospective study of 20 patients with high-cervical intramedullary spinal cord tumors (IMSCTs) and found that, while unimpaired D-wave monitoring reliably predicts preserved gross-motor function, it fails for distal upper extremity fine-motor/complex function. They reported that permanent deterioration in complex distal upper extremity function (15%) was best predicted by multimodal IONM with 43% sensitivity and 79% specificity.

Ilhan et al. (2024) [17] demonstrated that pre- and intraoperative neurophysiological alterations (MEPs, SSEPs) are strongly associated with risk of neurological deterioration 3 months after surgery. Their machine learning model predicted clinical outcome with 84% accuracy. The unique advantage of D-wave monitoring is its resistance to anesthetic effects and muscle relaxants, providing reliable monitoring of motor pathways even under deep anesthesia.

Technical considerations for optimal D-wave monitoring include electrode placement at the rostral and caudal ends of the surgical field, continuous monitoring throughout tumor resection, immediate surgical pause if the D-wave amplitude decreases by more than 50%, and correlation with other monitoring modalities for comprehensive assessment. The integration of D-wave monitoring with conventional IONM modalities has significantly improved the safety of intramedullary tumor surgery, allowing for maximal safe resection while minimizing neurological morbidity.

#### 3.2.5. IONM in Post-Traumatic and Osteoporotic Vertebral Fractures

The application of IONM in the surgical management of vertebral fractures, both post-traumatic and osteoporotic, has shown promising results. Ushirozako et al. [21] conducted a prospective multicenter observational cohort study of 350 consecutive patients with traumatic spinal injury and found that TcMEP monitoring showed 88% sensitivity and 90% specificity, though with a low positive predictive value (18.4%). For osteoporotic compression fractures requiring surgical intervention, IONM has shown value in procedures such as vertebroplasty and kyphoplasty. Although these procedures are generally considered low-risk, neurological complications can occur due to cement leakage or fragment displacement. The use of triggered EMG during pedicle screw placement in osteoporotic vertebrae is particularly valuable, as the altered bone architecture increases the risk of pedicle breach. Calancie et al. (2014) conducted two prospective, blinded, randomized studies (Part 1 and Part 2) [16,27] involving 71 patients (802 screws in Part 1 and 820 pedicle tracks tested in Part 2) and found that their novel method (four-pulse train in the pedicle track, leg EMG) accurately predicts medially malpositioned thoracic screws with 100% sensitivity for ≥2 mm encroachment. They also demonstrated that providing intraoperative feedback from the IONM test significantly reduced clinically relevant thoracic pedicle screw medial malpositioning (*p* = 0.02). Technical considerations for IONM in vertebral fracture surgery include baseline recordings before reduction maneuvers, continuous monitoring during reduction and fixation, special attention during cement injection in vertebroplasty/kyphoplasty, and lower thresholds for triggered EMG in osteoporotic bone. The integration of IONM in the surgical management of vertebral fractures has contributed to improved safety and outcomes in this challenging patient population.

### 3.3. Impact on Surgical Decision-Making and Long-Term Outcomes

Several studies reported that IONM alerts led to changes in surgical strategy. CreveCoeur et al. [23] found that, in 66 pediatric patients whose surgery was aborted due to IONM signal loss, nearly 75% had monitorable IONM signals at repeat OR within 2 weeks. All patients had full neurologic recovery, highlighting the value of responding to IONM alerts. Studies with long-term follow-up demonstrated that the neurological benefits of IONM persisted over time. Kim et al. [15] conducted a retrospective review with historical controls of 196 patients (132 IONM, 64 non-IONM) undergoing anterior cervical discectomy with fusion (ACDF) for ossification of the posterior longitudinal ligament (OPLL) and found that postoperative neurological deficit rates were significantly lower in the IONM group (3.79%) versus the non-IONM group (14.06%).

## 4. Results: Cost-Effectiveness Analysis

### 4.1. Study Characteristics

Several high-quality studies have focused on the economic analysis of IONM in spinal surgery. Ney et al. (2013) [28] conducted a detailed cost–benefit analysis using a decision model, while Charalampidis et al. (2020) [24] provided a comprehensive review of the economic aspects of IONM, including a dedicated section on cost-effectiveness and a table summarizing significant studies on the topic. Additional economic insights have been provided by Ney et al. (2015) [29], Kim et al. (2025) [18], and Fehlings et al. (2024) [26].

### 4.2. Economic Outcomes

#### 4.2.1. Direct Costs of IONM

Ney et al. (2013) reported a direct cost of USD 1535 per operation for IONM based on 2009 Medicare reimbursement rates [28]. This figure provides an important benchmark for evaluating the economic impact of IONM. Ajiboye et al. (2017) [9] referenced a study by Traynelis et al. (2012) [30] that estimated the hourly cost of IONM at USD 633.32, with a total cost of USD 1,024,754 in 2011 US dollars for Medicare reimbursement.

#### 4.2.2. Cost Savings

Ney et al. (2013) found that IONM saved USD 23,189 per case for the reference scenario of 50-year-old patients with a 5% neurological complication rate, considering lifetime costs after neurological deficit [28]. This saving was based on a prevention rate of 52.4% given an IONM alert, with 94.3% sensitivity and 95.6% specificity. Ney et al. (2015) analyzed a large national database (NIS) and found that, while IONM was associated with higher initial hospital costs, after adjustment for multiple factors, the difference was substantially reduced (9% increase) [29]. Additionally, IONM was associated with reduced length of stay. The study concluded that IONM was associated with better clinical outcomes with some increase in hospital costs among those discharged for simple spinal fusions and laminectomies. Charalampidis et al. (2020) cited studies such as Sala et al. (2007), which estimated that IONM would be cost-effective if costs did not exceed USD 977 per procedure in scoliosis surgery [24,31]. They also discussed studies with contrasting results, such as Traynelis et al., which found no economic benefit in the routine use of IONM in simple cervical procedures. Kim et al. (2025) conducted a prospective observational study comparing the cost-effectiveness of extended endoscopic lumbar foraminotomy (EELF, which used IONM) with transforaminal lumbar interbody fusion (TLIF) [18]. The primary outcome was the cost per quality-adjusted life year (QALY) gained. The study concluded that EELF was significantly more cost-effective (USD 15,536.0/QALY) compared with TLIF (USD 32,869.4/QALY).

#### 4.2.3. Factors Influencing Cost-Effectiveness and Cost-Effectiveness Thresholds

Three key factors were identified as major determinants of the cost-effectiveness of IONM: (1) the baseline risk of neurological complications from surgery; (2) lifetime costs after neurological deficit; and (3) the ability to prevent neurological deficits after an IONM alert. These factors explain the variation in cost-effectiveness across different types of spinal procedures and patient populations. Ney et al. (2013) demonstrated that IONM remains cost-effective when the neurological complication rate from surgery exceeds 0.3% (*p* < 0.001) and the prevention rate after an IONM alert is greater than 14.2% (*p* = 0.02) [28]. Fehlings et al. (2024) cited both Sala et al. (2007) and Ney et al. (2013) in their clinical practice guidelines, indicating that IONM could be cost-effective, especially if the baseline risk of the procedure is above a certain threshold (e.g., 0.3% according to Ney et al.) [26,28,31]. However, they acknowledged that evidence on resource requirements and costs is limited.

#### 4.2.4. Cost-Effectiveness by Surgery Type

*Deformity Surgery*: Given the high neurological complication rate in deformity surgery and the high efficacy of IONM in this context (as demonstrated by Kundnani et al. [19] with 100% sensitivity for NMEP), IONM is highly cost-effective in this type of surgery.

*Tumor Surgery*: In spinal tumor surgery, where the neurological complication rate can be significant, IONM demonstrates favorable cost-effectiveness with significant savings, especially considering the lifetime costs associated with permanent neurological deficits. Ilhan et al. [17] and Cabañes-Martínez et al. [22] both demonstrated the clinical value of IONM in tumor surgery, which translates to economic benefits.

*Cervical Spine Surgery*: In cervical spine surgery, with varying neurological complication rates, IONM’s cost-effectiveness depends on the specific procedure and patient risk factors. El Choueiri et al. [11] suggested in their discussion that “developing clinical algorithms to identify appropriate candidates for IONM could improve cost-effectiveness by directing its use to high-risk procedures”. Ajiboye et al. [9] referenced a study by Traynelis et al. [30] that reported no persistent postoperative neurological deficits in a series of patients undergoing cervical decompression and reconstruction without IONM.

*Lumbar Spine Surgery*: Kim et al. [18] provided specific cost-effectiveness data for lumbar procedures, showing that EELF with IONM was significantly more cost-effective than TLIF.

### 4.3. Long-Term Economic Impact and Economic Models

The economic studies emphasized the long-term economic impact of IONM. While initial hospital costs may appear higher with IONM, as noted by Ney et al. [29], the actual cost of IONM should be compared with lifetime costs resulting from neurological complications. The prevention of permanent neurological deficits, especially in younger patients, can result in substantial savings in healthcare costs and productivity over a lifetime. Ney et al. [28] employed a sophisticated decision model based on IONM sensitivity and specificity, IONM cost, prevention rate given an IONM alert, neurological complication rates, and lifetime costs after neurological injury (direct healthcare costs and lost wages/benefits). Results were analyzed using Monte Carlo simulation with 10,000 replications, providing a robust statistical evaluation of the cost-effectiveness of IONM (Table 1; Table 2).

**Table 1:** Summary of key studies on intraoperative neuromonitoring (IONM) in spine surgery, detailing the study type, patient population, pathology, and surgical interventions.

Author	Year	Study Type	Number of Patients	Type of Pathology	Type of Intervention Performed
Kundnani [19]	2010	Analysis of prospectively collected IONM data	354 consecutive patients	Adolescent idiopathic scoliosis (AIS)	Corrective surgery for AIS (instrumented fusion)
Calancie (Part 1) [16]	2014	Prospective, blinded, randomized clinical study (Methods and alarm criteria)	71 patients (802 screws)	Patients requiring thoracic pedicle screws (T1-L1) for various disorders (e.g., scoliosis, kyphosis, fusion)	Implantation of thoracic pedicle screws (T1-L1)
Calancie (Part 2) [27]	2014	Prospective, blinded, randomized neuromonitoring study (Role of feedback)	71 surgical cases (820 pedicle tracks tested)	Patients requiring thoracic pedicle screws	Implantation of thoracic pedicle screws, with/without IONM feedback guiding trajectory
Raynor [4]	2016	Retrospective review	12,375 patients	All spinal pathologies (deformity, degenerative, pathologic disease, trauma)	All spinal surgical procedures (cervical, thoracic/thoracolumbar, lumbosacral)
Ajiboye [9]	2017	Systematic review and meta-analysis	26,357 patients (from 10 studies included in the meta-analysis)	Patients undergoing anterior cervical spine surgery (ACSS) for conditions such as myelopathy, radiculopathy, infection, tumor, trauma, OPLL	Anterior cervical spine surgery (ACSS), including anterior cervical discectomy and fusions (ACDFs) and corpectomies
Ibrahim [14]	2017	Retrospective data collection/analysis	121 patients	Various spinal pathologies requiring surgery (including tumor resection, ACDF, fusion, laminectomy across cervical, thoracic, lumbar regions)	Various spinal procedures (halo adjustment, corpectomy, ACDF, tumor resection, laminectomy, fusion, etc.)
Thirumala [12]	2017	Systematic review and meta-analysis	2102 patients with IS (from 12 studies; 8 for the meta-analysis)	Idiopathic scoliosis (IS)	Corrective spinal surgery for IS
Charalampidis [24]	2020	Narrative review	N/A (Review summarizes multiple studies with varied patient numbers, e.g., Hilibrand et al. 427 cases, Nuwer et al. 51,263 cases)	Various spinal conditions (degenerative cervical, deformity, tumors, degenerative lumbar)	Spine surgery
Kim [15]	2021	Retrospective review with historical controls	196 patients (132 IONM, 64 non-IONM) for ACDF only	Ossification of the posterior longitudinal ligament (OPLL), with/without myelopathy	Anterior cervical spine discectomy with fusion (ACDF)
Ushirozako [21]	2023	Prospective multicenter observational cohort study	350 consecutive patients (TcMEP derivation rate 94%)	Traumatic spinal injury (cervical, thoracic, lumbar)	Traumatic spinal injury surgery
Alvi [10]	2024	Systematic review and meta-analysis	99,937 patients (from 163 studies) in total across different modalities. SSEP: 16,310 (52 studies). MEP: 71,144 (68 studies). EMG: 7888 (16 studies). Multimodal: 17,968 (69 studies).	Various spinal pathologies (deformity, degenerative, tumors, trauma, conus/cauda equina injuries, etc.)	Spine surgery (various types for the included pathologies)
Cabañes-Martínez [22]	2024	Observational, descriptive, retrospective cohort study	91 patients (36 monitored, 55 unmonitored)	Intradural spinal tumors (extramedullary and intramedullary)	Surgical resection of intradural spinal tumors
CreveCoeur [23]	2024	Multicenter, multidisciplinary, retrospective study	66 pediatric patients (<18 years) whose surgery was aborted due to IONM signal loss	Pediatric spinal deformity (idiopathic, congenital, neuromuscular)	Pediatric spinal deformity surgery (posterior spinal instrumentation and fusion), aborted due to IONM loss
Fehlings [26]	2024	Clinical Practice Guideline (GRADE process)	Refers to Alvi et al. 2024 [10] meta-analysis data: 99,937 patients (164 studies)	Patients undergoing spine surgery, focus on “high risk” for ISCI (complex deformity, cord compression/myelopathy, intramedullary tumors, unstable fx, OPLL)	Spine surgery
Guzzi [25]	2024	Narrative review	N/A (Review article)	Various neurosurgical conditions (brain tumors, neurovascular, epilepsy, spinal, peripheral nerve)	Neurosurgery (brain, spinal, peripheral nerve)
Ilhan [17]	2024	Prospective cohort study (based on methods)	67 patients	Intradural spinal cord tumors (SCTs), including intramedullary and extramedullary	Surgical resection of intradural spinal cord tumors
Siller [20]	2024	Prospective assessment/study	20 patients	High-cervical intramedullary spinal cord tumors (IMSCTs) (above C4/5 level)	Microsurgical resection of high-cervical IMSCTs
Yu [2]	2024	Prospective, randomized, controlled study	46 patients (23 IONM, 23 non-IONM)	Cervical spondylosis requiring anterior cervical surgery (≤2 segments)	Anterior cervical spine surgery (ACSS), including ACDF
Bai [13]	2025	Retrospective analysis	127 patients (64 IONM group, 63 control group)	Lumbar degenerative diseases (lumbar spinal stenosis, lumbar disc herniation, lumbar degenerative spondylolisthesis)	Unilateral biportal endoscopic (UBE) lumbar spine surgery (decompression, discectomy, ULBD)
Burkhard [3]	2025	Retrospective, single-center cohort study	401 patients	Degenerative lumbar conditions requiring primary posterior lumbar interbody fusion (PLIF)	Primary posterior lumbar interbody fusion (PLIF) (open posterior decompression laminectomy and instrumented fusion with pedicle screw fixation)
El Choueiri [11]	2025	Meta-analysis	187,162 patients (21,686 with IONM, 165,476 without IONM) from 7 studies	Degenerative cervical spine disorders (spondylosis, OPLL, radiculopathy, myelopathy)	Degenerative cervical spine surgeries (e.g., ACDF, laminoplasty)
Kim [18]	2025	Prospective observational study	45 patients (23 EELF, 22 TLIF) completed 12-month follow-up (52 enrolled)	Lumbar foraminal stenosis with dominant unilateral radicular pain	Extended endoscopic lumbar foraminotomy (EELF) vs. transforaminal lumbar interbody fusion (TLIF)
Zhang [1]	2025	Retrospective study	1622 patients	Cervical spinal canal stenosis (disc degeneration, ligamentum flavum hypertrophy, OPLL)	Cervical spinal canal decompression surgery (anterior or posterior approach)

**Table 2:** Summary of the sensitivity, specificity, and monitoring modalities of intraoperative neurophysiological monitoring (IONM) across key studies in spine surgery.

Author	Year	IONM Sensitivity	IONM Specificity	Monitoring Modalities (SSEP, MEP, D-Wave, EMG, etc.)
Ajiboye [9]	2017	Pooled: 71% (CI: 48–87%) for ACSS. Unimodal: 68% (CI: 39–88%). Multimodal: 88% (CI: 4–100%).	Pooled: 98% (CI: 92–100%) for ACSS. Unimodal: 99% (CI: 97–100%). Multimodal: 92% (CI: 81–96%).	SSEP, MEP, EMG (Unimodal and Multimodal IONM mentioned; specific modalities from reviewed studies in Table 3 include SSEP, MEP, and EMG)
Alvi [10]	2024	SSEP: 71.4% (95% CI: 54.8–83.7). MEP: 90.2% (95% CI: 86.2–93.1). EMG: 48.3% (95% CI: 31.4–65.6). Multimodal: 83.5% (95% CI: 81–85.7). (SSEP and multimodal values from the Abstract, the Results section in the paper has slightly different values)	SSEP: 97.1% (95% CI: 95.3–98.3). MEP: 96% (95% CI: 94.3–97.2). EMG: 92.9% (95% CI: 84.4–96.9). Multimodal: 93.8% (95% CI: 90.6–95.9).	SSEP, MEP, EMG, D-waves, Multimodal IONM
Bai [13]	2025	N/A (Study does not provide TP/FP/FN/TN data for calculating sensitivity for neurological deficits)	N/A (Study does not provide TP/FP/FN/TN data for calculating specificity for neurological deficits)	Somatosensory-evoked potentials (SEPs), motor-evoked potentials (MEPs), free electromyography (freeEMG)
Burkhard [3]	2025	SSEP events for PMD: 13.6%. EMG events for PMD: 36.4%.	SSEP events for PMD: 96.0%. EMG events for PMD: 71.0%.	Free-run electromyography (EMG) and somatosensory-evoked potentials (SSEPs)
Cabañes-Martínez [22]	2024	N/A (Study reported predictive outcomes for specific cases rather than overall sensitivity percentages from a 2 × 2 table analysis: 3 deficits were predicted by IONM alerts)	N/A (Similar to sensitivity, specific percentage not calculable from provided data)	mMEPs (muscle motor-evoked potentials via tES), Epidural MEPs (D-wave), SSEPs (lower and upper limb), Bulbocavernous Reflex (BCR), Motor Root Mapping, free-run EMG
Calancie [27] (Part 1)	2014	100% for predicting medial screw malpositioning ≥2 mm using 4-pulse pedicle track stimulation and leg EMG (with 15 mA cutoff)	High (AUC = 0.93 for predicting medial malposition ≥2 mm). Specificity depends on cutoff (e.g., 90% for 10 mA cutoff; 74% for 15 mA cutoff based on stated false positive rates of 10% and 26%, respectively).	Novel method: 4-pulse train stimulation in the pedicle track with evoked EMG from leg muscles (Quads, TA, AbH). Comparison methods: screw stimulation with IC/Abs EMG. Standard SSEP and transcranial MEP also performed.
Calancie [16] (Part 2)	2014	100% (for the IONM test itself, for ≥2 mm medial malposition, as established in Part 1)	N/A (Refer to Calancie et al. 2014 [27] Part 1)	4-pulse stimulus trains in the pedicle track, evoked EMG from leg muscles (AbH, TA, Quads), SSEP, MEP
Charalampidis [24]	2020	Varies by modality and study (Data from Table 2): - MEP (cervical CSM): 71% (Clark et al.). - tcMEP (cervical): 100% (Hilibrand et al.). - SSEP (cervical): 25% (Hilibrand et al.). - MIONM (ACDF): 80% (Kim et al.). - MIONM (deformity): ~100% (Quraishi et al., Bhagat et al.).	Varies by modality and study (Data from Table 2): - MEP (cervical CSM): 94% (Clark et al.). - tcMEP (cervical): 100% (Hilibrand et al.). - SSEP (cervical): 100% (Hilibrand et al.). - MIONM (ACDF): 97% (Kim et al.). - MIONM (deformity): 84–93.3% (Quraishi et al., Bhagat et al.).	SSEP, MEP (tcMEP, D-wave), EMG (spontaneous and triggered), Multimodal IONM (MIONM)
CreveCoeur [23]	2024	N/A (Study on outcomes *after* IONM loss, not diagnostic accuracy of IONM itself)	N/A (Study on outcomes *after* IONM loss, not diagnostic accuracy of IONM itself)	SSEPs, MEPs
El Choueiri [11]	2025	N/A (Meta-analysis evaluated effect on complication rates, not the pooled diagnostic sensitivity of IONM. One included study, Kim et al. 2021 [15], reported a sensitivity of 84.2% for multimodal IONM)	N/A (Meta-analysis evaluated effect on complication rates, not the pooled diagnostic specificity of IONM. One included study, Kim et al. 2021 [15], reported a specificity of 93.7% for multimodal IONM)	SSEP, MEP (tcMEP), EMG, Multimodal IONM
Fehlings [26]	2024	Summarizes Alvi et al.: SSEP: 67.5% (or 71.4%). MEP: 90%. EMG: 48.3%. Multimodal: 91% (or 83.5%).	Summarizes Alvi et al.: SSEP: 96.8% (or 97.1%). MEP: 95.6% (or 96%). EMG: 92.9%. Multimodal: 93.8%.	SSEP, MEP, EMG, D-Wave, Multimodal
Guzzi [25]	2024	N/A (Review of techniques and warning criteria, e.g., SSEP: >50% ampl. reduction or >10% lat. increase; BAEPs: >50% wave V ampl. reduction or >0.5 ms lat. increase; VEPs >50% or >20% decrement)	N/A (Review of techniques and warning criteria)	ECOG, SEEG, EMG, SSEP, MEP, DCS, BAEPs, VEPs
Ibrahim [14]	2017	57.14%	98.20%	Multimodality: SSEPs (98.4%), TCMEPs (86.3%), EMG (90.2%), TOF (34.1%), EEG (19.5%), BAER (1.6%)
Ilhan [17]	2024	Combined MEPs–DW/SSEPs (any alert, patient level for clinical deterioration at 3 months): 95%. For individual limb deterioration (any alert): MEPs–DW 78%; SSEPs 42%; Combined 81%.	Combined Persistent MEPs–DW/SSEPs (patient level for clinical deterioration at 3 months): 84%. For individual limb deterioration (any alert): MEPs–DW 82%; SSEPs 83%; Combined 72%.	Multimodal IONM: SSEPs, MEPs (transcranial electrical stimulation for muscle MEPs and D-wave)
Kim [15]	2021	Multimodal IONM: 84.2% (including indeterminate warnings). tcMEP: 71.4%. EMG: 69.2%. SSEP: 0%.	Multimodal IONM: 93.7% (including indeterminate warnings). tcMEP: 94.1%. EMG: 99.1%. SSEP: 100%.	Multimodal IONM: tcMEP, SSEP, continuous EMG
Kim [18]	2025	N/A (IONM used in the EELF group, but the study is a cost-effectiveness comparison, not the diagnostic accuracy of IONM)	N/A (IONM used in the EELF group, but the study is a cost-effectiveness comparison, not the diagnostic accuracy of IONM)	For EELF: Multimodal IONM (fEMG, MEP, SSEP)
Kundnani [19]	2010	NMEP: 100%. SSEP: 51%. Combined SSEP + NMEP: 100%.	NMEP: 96%. SSEP: 100%. Combined SSEP + NMEP: 99%. (Abstract reports 98.5% for combined specificity)	Multimodal: SSEP and NMEP (neurogenic motor-evoked potential via transcranial electrical stimulation)
Raynor [4]	2016	N/A (Study focuses on false negative rates, i.e., failure of IOM to detect deficits, not overall diagnostic sensitivity)	N/A (Study focuses on false negative rates)	Multimodal IOM: SSEPs, DNEPs (descending neurogenic), MEPs (transcranial), DSEPs (dermatomal), spEMG (spontaneous), trgEMG (triggered)
Siller [20]	2024	D-wave for fine-motor/complex hand function: Low. Multimodal IONM (D-wave/mMEPs/EMG/SSEPs) for complex distal upper extremity function (NHPT): 43% (long-term deterioration).	Multimodal IONM (D-wave/mMEPs/EMG/SSEPs) for complex distal upper extremity function (NHPT): 79% (long-term deterioration).	Multimodal IONM: D-wave, mMEPs (muscular MEPs via TcMEP), free-running EMG (frEMG), SSEPs. Dorsal column mapping (DCM) also used.
Thirumala [12]	2017	Pooled mean 91% (95% CI: 34–100%) for TcMEP changes	Pooled mean 96% (95% CI: 92–98%) for TcMEP changes	Transcranial motor-evoked potential (TCMEP) monitoring (some studies also used SSEP)
Ushirozako [21]	2023	88% (also 87.5%) for TcMEP	90% (also 90.3%) for TcMEP	Multimodal IONM: TcMEP, SSEP, free-run EMG (study focused on TcMEP)
Yu [2]	2024	N/A (Study evaluated the IONM effect on dysphagia, not diagnostic accuracy for general neurological deficits)	N/A (Study evaluated the IONM effect on dysphagia, not diagnostic accuracy for general neurological deficits)	Multimode monitoring: motor-evoked potential (MEP), somatosensory-evoked potential (SEP), electromyography (EMG)
Zhang [1]	2025	High-risk group: 100%. Low-risk group: 91.7%.	High-risk group: 98.84%. Low-risk group: 98.79%.	Multimodal IONM: SSEP, MEP, EMG (TOF also mentioned for anesthesia)

**Table 3:** Summary of key findings from major studies on the clinical effectiveness, limitations, and outcomes associated with intraoperative neurophysiological monitoring (IONM) in spine surgery.

Author	Year	Key Findings
Ajiboy e [9]	2017	The risk of neurological injury after ACSS is low (0.64% weighted). Corpectomies may carry higher risk (1.02%) than ACDFs (0.20%). For ACDFs, no difference in neurological injury risk with/without IONM. Unimodal IONM has higher specificity than multimodal IONM.
Alvi [10]	2024	All neuromonitoring modalities (SSEP, MEP, EMG, multimodal monitoring) have diagnostic utility in detecting impending/incident intraoperative neurologic injuries, though accuracy differs. MEP showed the highest sensitivity (90.2%). SSEP showed high specificity (97.1%). EMG had lower sensitivity (48.3%). Multimodal monitoring showed high sensitivity (83.5%) and specificity (93.8%).
Bai [13]	2025	In the IONM group, 62.5% showed freeEMG stimulation; 15.6% had an MEP amplitude decrease. No significant SEP changes. Postop 24 h leg VAS lower in the IONM group ($*p*\<0.001$). IONM provides timely neurological function info, reduces invasiveness, and reduces early postop leg pain.
Burkhard [3]	2025	SSEP events associated with postoperative motor deficits (PMDs) (*p* = 0.043), EMG events were not. Persistent SSEP signal loss strongly predicted PMDs (OR 10.41). Overall test accuracy of IONM (SSEP and EMG) limited; IONM should be tailored to individual cases.
Cabañes-Martínez [22]	2024	IONM use was associated with improvements in Prolo, Brice–McKissock, and McCormick scales. Rate of new/worsened neurological deficits was 8.3% in the monitored group vs. 14.5% in the unmonitored group. IONM predicted deficits in 3 monitored patients with worsened outcomes. Six IONM events were resolved intraoperatively without deficit.
Calancie [27] (Part 1)	2014	Novel method (4-pulse train in the pedicle track, leg EMG) accurately predicts medially malpositioned thoracic screws (100% sensitivity for ≥2 mm encroachment). This method is superior to direct screw stimulation or using IC/Abs EMG. Optimal alarm criteria: 10 mA (lower) and 15 mA (upper) cutoffs.
Calancie [16] (Part 2)	2014	Providing intraoperative feedback from the novel IONM test (alarm ≤ 10 mA) significantly reduced clinically relevant (≥2 mm) thoracic pedicle screw medial malpositioning (*p* = 0.02). All 32 screws with ≥2 mm medial encroachment occurred when feedback was withheld or not acted upon. Traditional SSEP/MEP failed to detect these malpositioned screws.
Charalampidis [24]	2020	MIONM is generally effective for detecting perioperative neurological injury. SSEP can be delayed; MEPs are sensitive to anesthesia; EMG has high a false positive rate. Growing evidence supports IONM, but protocols for managing alerts are lacking. Controversy exists for routine use in noncomplex cervical procedures. MIONM well-supported for spinal tumor resection.
CreveCoeur [23]	2024	Nearly 75% of children with aborted spinal deformity surgery (due to IONM loss) had monitorable IONM signals at repeat OR within 2 weeks. Bilateral SSEP loss, combined bilateral SSEP/MEP loss, and delayed neurologic recovery (>72 h) linked to unmonitorable IONM at repeat surgery. All patients had full neurologic recovery.
El Choueiri [11]	2025	Pooled analysis showed no statistically significant protective effect of IONM in degenerative cervical spine surgery (OR = 0.90). Subgroup analysis suggested potential benefits when EMG is used with SSEP and MEP (OR = 0.39). Sample size significantly explained heterogeneity.
Fehlings [26]	2024	Recommends IONM for high-risk spine surgery patients (Strong recommendation, Low-quality evidence). Suggests proactive identification of “high-risk” patients, multidisciplinary team discussions, and intraop protocol with IONM (Weak recommendation, Very-Low-quality evidence). Developed AO Spine-PRAXIS Care Pathway for ISCI.
Guzzi [25]	2024	IONM is crucial for enhancing safety and precision in neurosurgery. Requires a detailed anesthetic plan (TIVA preferred). Discusses IONM applications in intracranial tumor resection, neurovascular surgery, epilepsy surgery, spinal surgery, and peripheral nerve surgery. Acknowledges limitations like false positives.
Ibrahim [14]	2017	IONM had low sensitivity (57.14%) in this cohort, potentially producing excessive false negatives. Specificity was high (98.20%). Authors suggest surgeons rely on clinical/surgical judgment.
Ilhan [17]	2024	Pre- and intraoperative neurophysiological alterations (MEPs, SSEPs) are strongly associated with risk of neurological deterioration 3 months after surgery. Transient intraoperative MEP/SSEP alterations did not pose a risk of neurological deterioration. Machine learning model predicted clinical outcome with 84% accuracy.
Kim [15]	2021	Postoperative neurological deficit rates significantly lower in the IONM group (3.79%) vs. the non-IONM group (14.06%). Use of IONM (OR: 0.139) associated with postoperative neurological complications. Multimodal IONM may be useful in ACDF for high-risk OPLL. No SSEP warnings observed.
Kim [18]	2025	EELF was significantly more cost-effective than TLIF. EELF had shorter operating times, less blood loss, and shorter hospital stays. No significant difference in clinical outcomes at 12 months. fEMG events in 52.2% of EELFs. MEP decline in 21.7%. No SSEP declines.
Kundnani [19]	2010	NMEP is superior to SSEP for identifying evolving spinal cord injury (sensitivity 100% vs. 51%). Specificity of SSEP (100%) is higher than that of NMEP (96%). Combined (SSEP + NMEP) monitoring achieved 100% sensitivity and 99% specificity. Multimodality monitoring should be standard of care.
Raynor [4]	2016	In total, 45 of 12,375 patients (0.36%) had false negative IOM outcomes. Most false negatives involved spEMG (48.8%). Eight patients (0.064%) had permanent neurologic deficits not detected by IOM. Undetected deficits have a higher risk of being permanent. Nerve root monitoring (spEMG) failures were most common.
Siller [20]	2024	Unimpaired D-wave reliably predicts preserved gross-motor function but fails for distal upper extremity fine-motor/complex function in high-cervical IMSCT surgery. Permanent deterioration in complex distal upper extremity function (15%) best predicted by multimodal IONM (sens, 43%; spec, 79%). Underlines the importance of multimodal IONM for fine-motor/complex hand function.
Thirumala [12]	2017	TcMEP monitoring is a highly sensitive (91%) and specific (96%) test for detecting new spinal cord injuries in IS surgery. A patient with a new neurological deficit from IS surgery was 250 times more likely to have TcMEP changes. AUC for TcMEP was 0.98.
Ushirozako [21]	2023	TcMEP monitoring showed 88% sensitivity and 90% specificity. Low PPV (18.4%). Neurological complication rate was 2.3%. Most common TcMEP alert timing: during decompression (40%). Single usage of TcMEP not recommended due to a low PPV.
Yu [2]	2024	IONM significantly reduced dysphagia 12 weeks post-ACSS (13.0% vs. 43.5%). No significant difference at 3rd day or 6th week post-surgery. Significantly less delayed swallowing trigger and residue with IONM at 12 weeks.
Zhang [1]	2025	Ligamentum flavum hypertrophy, OPLL, and preoperative mJOA < 12 are high-risk factors for intraoperative alarms. Sensitivity and PPV higher in the high-risk group. Irreversible IONM alarms correlated with poorer mJOA recovery. Reversibility of alarms is a principal predictor of outcomes.

## 5. Discussion

### 5.1. Clinical Implications

The findings of this systematic review have several important clinical implications. The first is the importance of the routine use of IONM in spinal surgery, particularly for high-risk procedures such as deformity correction and tumor resection. The substantial reduction in neurological deficits translates to meaningful improvements in patient outcomes and quality of life [14,15,17,19,26]. While IONM has demonstrated utility during pedicle screw insertion, its value is substantially more pronounced during deformity correction maneuvers, where neurological structures face greater biomechanical stresses and potential compromise. A misplaced pedicle screw may remain clinically silent without causing a neurological deficit, whereas the forceful manipulation and realignment of spinal elements during deformity correction presents a more significant neurological risk profile that benefits from real-time monitoring. In addition, the combination of SSEPs, MEPs, and EMG provides the most comprehensive assessment of neurological function during surgery, compensating for the limitations of each individual modality [20,30,32].

This review shows the importance of IONM in guiding intraoperative decision-making. The specific findings on D-wave monitoring for intramedullary tumors provide valuable guidance for this challenging surgical scenario. The fact that IONM alerts led to surgical modifications in many cases demonstrates its role not just as a warning system but as an active guide for surgical strategy. The high correlation between D-wave preservation and postoperative motor function makes this modality particularly valuable for tumor resection. Data on IONM in vertebral fractures expand the application of this technology to an important and common clinical scenario, providing guidance for both post-traumatic and osteoporotic fractures.

### 5.2. Economic Implications

The economic analysis provides compelling evidence that IONM is not only clinically effective but also economically sound. In a common global society where more and more attention is being paid to hospital costs and the economic aspect of hospitals [33,34], the finding that IONM is cost-effective when the neurological complication rate exceeds 0.3% means that it is economically justified for the vast majority of spinal procedures. The substantial savings reported (USD 23,189 per case on average, and over USD 1 million for high-risk procedures) highlight the economic value of preventing neurological complications. These savings benefit not only healthcare systems but also society as a whole by reducing disability and preserving productivity. The identification of specific thresholds for cost-effectiveness provides practical guidance for clinical decision-making. Surgeons and healthcare administrators can use these thresholds to determine when IONM is economically justified based on the type of procedure and patient characteristics. The economic analysis of D-wave monitoring for intramedullary tumors and IONM for vertebral fractures provides specific guidance for these clinical scenarios, allowing for targeted application of resources where they provide the greatest value.

### 5.3. Medico-Legal Issue of Intraoperative Neurophysiological Monitoring

Intraoperative neurophysiological monitoring (IONM) presents significant medico-legal considerations for spine surgeons, as evidenced by recent studies. Hatef (2020) analyzed 26 medical malpractice cases related to IONM in spinal fusion surgeries, finding that defendant verdicts were most common (54%), followed by settlements (27%) and plaintiff verdicts (19%) [33]. Notably, settlements averaged USD 7,575,000, while plaintiff verdicts averaged USD 4,180,213. Litigation was based on failure to monitor (54%) and negligent monitoring (46%), suggesting that spine surgeons face malpractice risks from both the non-use of IONM when indicated and improper interpretation of monitoring findings. Taylor (2025) surveyed 188 neurosurgeons and found that 45% reported using IONM specifically for medico-legal reasons, highlighting its role as a defensive medicine practice [35]. Despite this, opinions on IONM’s necessity varied considerably, with 55% disagreeing and 32% agreeing it was necessary, indicating a lack of consensus on its status as a standard of care for pedicle screw placement. The utilization patterns of IONM have evolved over time, with Ajiboye (2017) documenting an increase from 27% in 2005 to 46.9% in 2011 in scoliosis surgery [9]. From a cost-effectiveness perspective, Ament (2023) analyzed 17,929 spine patients and found that, while IONM added approximately USD 1547 to index surgery costs, it was associated with significantly lower neurological deficit rates (0.3% vs. 4.1%, *p* < 0.01) [36]. This translated to IONM being cost-effective from a health system perspective (ICER USD 60,734 per QALY) and dominant from a societal perspective. However, Krause (2020) found that neuromonitoring for lumbar discectomy increased operating room times and costs without discernible differences in neurological outcomes, suggesting context-specific utility [37]. Similarly, Burkhard (2025) concluded that, while SSEP events were predictive of postoperative motor deficits, the overall predictive accuracy of IONM was limited, recommending its application be tailored to individual cases [3]. These findings collectively suggest that the medico-legal landscape surrounding IONM is complex, with its use influenced by both clinical evidence and liability concerns. Given the substantial financial implications of malpractice settlements and the increasing utilization trends, surgeons should carefully document their decision-making process regarding IONM use or non-use in each case. Furthermore, the development of clear, evidence-based guidelines for IONM application could help standardize practice and potentially reduce the medico-legal vulnerability while optimizing patient outcomes.

### 5.4. Future Directions

Several areas warrant further investigation. First, there is a need for more prospective studies and, where ethically feasible, randomized controlled trials to strengthen the evidence base for IONM. Research should focus on refining IONM techniques and protocols to improve sensitivity and specificity, particularly for challenging scenarios such as complex deformity correction and tumor resection. In addition, economic analyses should be updated as technology and healthcare costs evolve and should be expanded to include a wider range of healthcare settings and payment models. The integration of IONM with other advanced technologies, such as navigation and robotics, represents an exciting frontier that may further enhance the safety and efficacy of spinal surgery. Standardized protocols for responding to IONM alerts should be developed and validated to ensure optimal patient outcomes when neurological compromise is detected.

### 5.5. Limitations of the Study

This systematic review has several limitations. First, most of the included studies were observational, with few randomized controlled trials. This reflects the ethical challenges of randomizing patients to no monitoring in high-risk procedures. Second, there was heterogeneity in the definition of neurological deficits and the methods of IONM across studies. We attempted to account for this in our analysis, but it may affect the precision of our estimates. Third, the economic analysis was based on models and assumptions that may not fully capture the complexity of real-world healthcare costs and outcomes. However, the consistency of findings across different models and sensitivity analyses suggests that the conclusions are robust. Fourth, most studies were conducted in high-resource settings, and the findings may not be fully generalizable to low-resource environments where the costs and availability of IONM may differ.

## 6. Conclusions

This comprehensive systematic review provides strong evidence that intraoperative neurophysiological monitoring significantly improves clinical outcomes in spinal surgery. The diagnostic accuracy varies by modality, with MEP showing the highest sensitivity (90.2%) and SSEP demonstrating high specificity (97.1%). Multimodal monitoring provides the most comprehensive assessment, while D-wave monitoring offers particular benefits for intramedullary tumor surgery. From an economic perspective, IONM is cost-effective in most clinical scenarios, particularly when the neurological complication rate exceeds 0.3%. The average saving of USD 23,189 per case, and potential savings exceeding USD 1 million for high-risk procedures, demonstrates the substantial economic value of preventing neurological complications.

These findings support the routine use of IONM in contemporary spinal surgery, especially for complex and high-risk procedures. The clear separation of clinical and economic analyses in this systematic review provides a comprehensive understanding of both aspects of IONM, guiding evidence-based clinical practice and resource allocation.

## Abbreviations

The following abbreviations are used in this manuscript:

IONM	Intraoperative Neurophysiological Monitoring
SSEP	Somatosensory-Evoked Potential
MEP	Motor Evoked Potential
EMG	Electromyography
MIONM	Multimodal Intraoperative Neurophysiological Monitoring
NMEP	Neurogenic Motor-Evoked Potential
TcMEP	Transcranial Motor-Evoked Potential
ACSS	Anterior Cervical Spine Surgery
ACDF	Anterior Cervical Discectomy and Fusion
PLIF	Posterior Lumbar Interbody Fusion
IMSCT	Intramedullary Spinal Cord Tumor
OPLL	Ossification of the Posterior Longitudinal Ligament
PMD	Postoperative Motor Deficit
